# Evaluation of the accuracy of diagnostic scales for a syndrome in Chinese medicine in the absence of a gold standard

**DOI:** 10.1186/s13020-016-0100-2

**Published:** 2016-07-28

**Authors:** Xiao Nan Wang, Vanessa Zhou, Qiang Liu, Ying Gao, Xiao-Hua Zhou

**Affiliations:** 1 School of Statistics, Renmin University of China, Beijing, 100872 China; 2 Department of Biostatistics, School of Public Health, University of Washington, Seattle, WA 98195 USA; 3 UW Autism Center, University of Washington, Seattle, WA 98105 USA; 4 TCM International Cooperation Center of China, Beijing, 100101 China; 5 Department of Neurology, Dongzhimen Hospital of Beijing University of Chinese Medicine, Beijing, 100700 China

**Keywords:** CM, Sensitivity, Specificity, Diagnostic scale, Syndrome, Bayesian method, Without a gold standard

## Abstract

**Background:**

The concept of syndromes *(zhengs)* is unique to Chinese medicine (CM) and difficult to measure. Expert consensus is used as a gold standard to identify *zhengs* and evaluate the accuracy of existing diagnostic scales for *zhengs*. But, the use of expert consensus as a gold standard is problematic because the diagnosis of *zhengs* by expert consensus is not 100 % accurate. This study aimed to evaluate the accuracy of standardized diagnostic scales for a syndrome *zhengs* in the absence of a gold standard, with application to internal wind (nei feng) syndrome in ischemic stroke patients.

**Methods:**

A total of 204 participants (age 41–84 years) with ischemic stroke were assessed by the stroke syndrome differentiation diagnostic criterion (SSDC), ischemic stroke TCM syndrome diagnostic scale (ISDS), and expert syndrome differentiation (ESD). The diagnostic tests and data collection process were conducted over a 10-month period (February 2008 to November 2008) in 10 hospitals across nine cities in China. The Bayesian method was used to estimate the accuracy of the SSDC, ISDS, and ESD.

**Results:**

For internal wind syndrome, the estimated sensitivities and specificities of the SSDC, ISDS, and ESD without use of a gold standard were respectively: $\widehat{Se}_{1}=0.687$, $\widehat{Sp}_{1}=0.776$; $\widehat{Se}_{2}=0.884$, $\widehat{Sp}_{2}=0.875$; and $\widehat{Se}_{3}=0.813$, $\widehat{Sp}_{3}=0.922$

**Conclusion:**

After adjusting for imperfect gold standard bias, we found that both the sensitivity and specificity of the ISDS were higher than those of the SSDC for diagnosis of internal wind syndrome in ischemic stroke patients.

**Electronic supplementary material:**

The online version of this article (doi:10.1186/s13020-016-0100-2) contains supplementary material, which is available to authorized users.

## Background

The concept of syndromes (*zhengs*) is unique to Chinese medicine (CM). Syndromes are identifiable from a holistic understanding of a patient’s clinical presentation using the four CM diagnostic methods: observation, listening/smelling, questioning, and pulse analyses [1]. Identification of a syndrome can differ from one CM practitioner to another because of varying medical experience and other related factors. In recent years, the CM community has developed several standardized diagnostic scales for syndromes [2–5]. The accuracies of these scales have been assessed by the diagnostic opinion of CM practitioners as the gold standard. However, an expert diagnosis is largely dependent on clinical experience and educational background, leading to different syndrome differentiation for the same patient by different expert CM practitioners. This results in biased estimates for the accuracy of diagnostic scales because the expert syndrome differentiation (ESD) is imperfect. Such bias is called an imperfect gold standard bias [6, 7]. If the diagnostic test and imperfect gold standard are conditionally independent of the true disease status, the sensitivity and specificity of the diagnostic test are underestimated. However, if the diagnostic test and imperfect gold standard are conditionally dependent, the estimated sensitivity and specificity of the diagnostic test can be biased in either direction. The direction of the bias is determined by the degree to which the diagnostic tests and imperfect gold standard misclassify the same patients. When this tendency is slight, the accuracy of the diagnostic test is generally underestimated; when the tendency is strong, the accuracy of the diagnostic test is generally overestimated [6].

In recent years, several statistical methods have been developed to correct imperfect gold standard bias. Hui and Waiter [8] developed a model for two diagnostic tests within two populations and introduced a maximum likelihood approach when assuming the existence of two populations strata with different prevalence rates. In that model, they also assumed that the two tests were conditionally independent. However, the assumption of conditional independence may not be realistic in some applications owing to some common factors that can influence both diagnostic tests and true disease status. Sinclair and Gastwirth [9] extended the Hui and Waiter model to allow for conditional dependence. Espeland and Handelman [10] and Yang and Becker [11] proposed latent class modeling for conditional dependence, Qu et al. [12], Hadgu and Qu [13] proposed random effects models, and Albert and Dodd [14] developed latent class modeling approaches for binary tests. Pepe and Janes [15] discussed the latent class analysis method when assessing the multiple diagnostic tests without a gold standard, and concluded that a latent class model required careful justification of assumptions made about the conditional dependence structure. These researchers also stressed that a formal clinical definition of the disease should be given before evaluating the accuracy of diagnostic tests with the latent class method. Only when the disease has been clearly defined can the estimated parameters be meaningful for diagnostic tests; otherwise, the results of the estimators were meaningless. The above-mentioned methods used the frequentist approach to estimate the parameters in the model when the diagnostic tests were conditionally independent, given the true disease status or given the true disease status and a random effect.

Joseph et al. [16] used Bayesian methods to assess the accuracy of diagnostic tests under conditional independence without a gold standard. Dendukuri [17], Georgiadis et al. [18], and Branscum et al. [19] developed Bayesian models to evaluate the accuracy of diagnostic tests with two conditionally dependent tests. These methods have been widely used for estimation of the accuracy of diagnostic tests without a gold standard in Western medicine research [20–27]. However, they have not been applied for estimation of the accuracy of diagnostic tests for CM syndromes. This study aimed to evaluate the accuracy of standardized diagnostic scales for a syndrome in the absence of a gold standard, with application to internal wind (nei feng) syndrome in ischemic stroke patients.

## Methods

### Study design and approval

In this study, we evaluated the accuracy of the stroke syndrome differentiation diagnostic criterion (SSDC), ischemic stroke TCM syndrome diagnostic scale (ISDS), and ESD for detecting “internal wind” in ischemic stroke patients, without assuming that the ESD is the gold standard. We mainly focused on comparing the accuracy of the two diagnostic scales (SSDC and ISDS).This study used data from the second round of a diagnostic test study of the ISDS. The diagnostic test and data collection process were performed over a 10-month period (February 2008 to November 2008), after receiving approval (ECSL-BDY-2008-012) from the Ethics Committee of the Dongzhimen Hospital of Beijing University of Chinese Medicine (Additional files 1 and 2).

### Inclusion and exclusion criteria

Individuals who had a confirmed diagnosis of acute ischemic stroke by computed tomography and magnetic resonance imaging examinations, were aged between 35 and 85 years, and were informed of the objectives and research procedures of the study (details of study please see Additional file 3) and provided signed consent forms themselves (consent forms please see Additional file 4) were selected as the participants in this study [5]. We excluded individuals with the following symptoms: transient ischemic attack; cerebral hemorrhage or subarachnoid hemorrhage; stoke caused by brain tumor, traumatic brain injury, or blood disease; severe heart, liver, kidney, or hematopoietic system comorbidity and complication; mental disorder or severe dementia; and severe aphasia that could affect data collection [5].

### Study subjects

The final data set comprised 204 patients from 10 hospitals across nine cities in China [4, 5]. All of the participants (age 41–84 years ) were diagnosed with ischemic stroke. The mean age of the patients was 65 years, and the mode age was 74 years. The subjects were diagnosed as “0” or “1” by each of the SSDC, ISDS, and ESD. The detailed results of the cross-classification of the three diagnostic tests for internal wind syndrome in the 204 ischemic stroke participants are shown in Table 1. The CM syndrome factor scales (SSDC and ISDS) of the symptoms and signs, and the ESD were separately completed on the same day. In this study, an expert was defined as a physician, who had the clinical title of deputy director or above and also had more than 10 years of clinical work experience in diagnosing and curing stroke disease with traditional CM.

**Table 1:** Cross-classified test results of $$T_{1} $$, $$T_{2}$$ and $$T_{3}$$ for internal wind syndrome

$T_{1}$	$T_{2}=1$		$T_{2}=0$	
	$T_{3}=1$	$T_{3}=0$	$T_{3}=1$	$T_{3}=0$
$T_{1}=1$	69	19	7	12
$T_{1}=0$	32	9	5	51

### CM syndrome factor scales and syndrome differentiation

The SSDC and ESD were used to diagnose the status of a patient in place of a gold standard, before the development of the ISDS. The SSDC was the first recognized scale for diagnosing a CM syndrome in ischemic stroke patients, and has been widely used since its publication in 1994 [2, 3]. The development of the ISDS was based on the SSDC. Essentially, the ISDS is an updated version of the SSDC [3], and was first developed in 2007. The simple process for developing the ISDS has been described in the published literature [3, 4]. Briefly, the ISDS was developed from a two-round Delphi study, which generated a pool of draft items with 288 items in six syndrome factor dimensions [4]. From this pool of items, six syndrome factor diagnostic scales were constructed according to logistic regression functions and receiver-operating curve analysis. Each syndrome factor diagnostic scale consisted of 10–20 “yes” or “no” statements. The ESD was completed by three senior physicians with over 10 years of work experience [4, 5]. When the practitioners failed to reach a unanimous decision about a patient’s diagnosis, the majority opinion was used.

### Statistical methods

Descriptive statistics were utilized to summarize the characteristics of the subjects in the data set. The latent class model was fitted to the results of the SSDC, ISDS, and ESD for the ischemic stroke patients when a gold standard was not available. The Bayesian method was used to estimate the sensitivity and specificity for every CM diagnostic scale. We followed the guidelines for reporting Bayesian analyses in biomedical journals, as described by Lang and Altman [28].

Using the reporting guidelines, we first described the general Bayesian statistical model. Next, we specified the pre-trial probabilities (prior distributions) for the parameters in the proposed model based on the data we wanted to analyze and also explained how the prior distributions were selected. Subsequently, we used Markov chain Monte Carlo (MCMC) techniques to obtain the Bayesian estimated parameters, based on the posterior distribution. The median and credibility interval were used as the posterior summary measures in this study. Finally, we illustrated the sensitivity of the analyses to different prior distributions in the Bayesian model.

IBM SPSS Statistics for Windows [version: 21.0; IBM Crop; NY] was utilized for the descriptive statistics. WinBUGS software [version: 1.4.3; BUGS project; UK] was used for the Bayesian data analysis (WinBUGS code for this study could be found in Additional file 5). A detailed description of the proposed Bayesian method for evaluating the accuracy of the diagnostic tests without a gold standard is given as below.

### Notation

Let $T_{1}$, $T_{2}$, and $T_{3}$ denote the diagnostic results of the two CM diagnostic tests (SSDC and ISDS) and ESD for one syndrome factor in ischemic stroke patients, where $T_{1}$, $T_{2}$, and $T_{3}=0,1$, with “1” indicating the presence of the syndrome factor and “0” indicating the absence of the syndrome factor. Let *D* denote the true status of the syndrome factor in an ischemic stroke patient, which is not observed in the study. The parameters of interest include: the prevalence of the syndrome factor in the population, $\pi$, defined as $\pi =P(D=1)$; the sensitivity of the *i*th diagnostic test in detecting the syndrome factor, $Se_{i}$, defined as $Se_{i}=P(T_{i}=1|D=1)$; and the specificity of the *i*th diagnostic test for detecting the syndrome factor, $Sp_{i}$, defined as $Sp_{i}=P(T_{i}=0|D=0)$, where $i=1,2,3$.

### Bayesian model

Assume that there are *n* participants in the sample and three test results for every subject. We represent the observed data as $Y=(Y_{t_{1},t_{2},t_{3}})$, where $Y_{t_{1},t_{2},t_{3}}$ is the number of subjects with $T_{1}=t_{1}$, $T_{2}=t_{2}$, and $T_{3}=t_{3}$; here $t_{1},t_{2},t_{3}=0,1$. For example, $Y_{111}$ denotes the number of subjects whose diagnostic results for all three tests indicate that the syndrome factor is present. Correspondingly, $p_{t_{1},t_{2},t_{3}}$ represents the joint probability of the outcome $(T_{1}=t_{1},T_{2}=t_{2},T_{3}=t_{3})$, which is defined as follows:

1 $$\begin{aligned} p_{t_{1},t_{2},t_{3}} & = {} P(T_{1}=t_{1},T_{2}=t_{2},T_{3}=t_{3})\nonumber \\ &=  {} P(T_{1}=t_{1},T_{2}=t_{2},T_{3}=t_{3}|D=1)\times P(D=1)\nonumber \\ & \quad +  {} P(T_{1}=t_{1},T_{2}=t_{2},T_{3}=t_{3}|D=0)\times P(D=0)\nonumber \\& = {} P(T_{1}=t_{1},T_{2}=t_{2},T_{3}=t_{3}|D=1)\times \pi \nonumber \\ & \quad +  {} P(T_{1}=t_{1},T_{2}=t_{2},T_{3}=t_{3}|D=0)\times (1-\pi ). \end{aligned}$$

Among the three tests in this study, the first two tests represent the diagnostic results of the SSDC and ISDS, respectively, and the last test represents the diagnostic result of the expert opinion, called the ESD. Since the two diagnostic scales consist of standardized questionnaires, while the ESD is based on the individual opinion of expert CM practitioners, it is reasonable to assume that the CM expert and the diagnostic scales err independently (i.e., they are conditionally independent, given the true CM syndrome status). Nevertheless, the two diagnostic scales do not err independently (i.e., they are conditionally dependent, given the true CM syndrome status). Such dependence is measured by the conditional dependence correlations, given the true CM syndrome status. Hence, we assume that $T_{3}$ is independent of $T_{1}$ and $T_{2}$ conditional on *D*, while we allow $T_{1}$ and $T_{2}$ to be conditionally dependent, given *D*. Let $C_{+}$ and $C_{-}$ denote the covariance between $T_{1}$ and $T_{2}$ among the CM syndrome positive and negative individuals, respectively. In other words, $C_{+}=cov(T_{1},T_{2}|D=1)$ and $C_{-}=cov(T_{1},T_{2}|D=0)$. Such a model has also been studied by Dendukuri and Joseph [17]. To present the Bayesian method, we need to compute the likelihood function of the observed data. Note that we can respectively write $P(T_{1}=t_{1},T_{2}=t_{2}|D=1)$ and $P(T_{1}=t_{1},T_{2}=t_{2}|D=0)$ as follows:

$$\begin{aligned} P(T_{1}=t_{1},T_{2}=t_{2}|D=1)=\prod \limits _{i=1}^{2}Se_{i}^{t_{i}}(1-Se_{i})^{(1-t_{i})}+(-1)^{t_{1}+t_{2}}C_{+}, \\ P(T_{1}=t_{1},T_{2}=t_{2}|D=0)=\prod \limits _{i=1}^{2}Sp_{i}^{(1-t_{i})}(1-Sp_{i})^{t_{i}}+(-1)^{t_{1}+t_{2}}C_{-}. \end{aligned}$$

Consequently, we can rewrite the joint probability of the outcome (*T*_1_ = *t*_1_, *T*_2_ = *t*_2_, *T*_3_ = *t*_3_) as follows:

2 $$\begin{aligned}
p_{t_{1},t_{2},t_{3}} &=  P(T_{1}=t_{1},T_{2}=t_{2}|D=1)\times
P(T_{3}=t_{3}|D=1)\times \pi \nonumber \\
& \quad + P(T_{1}=t_{1},T_{2}=t_{2}|D=0)\times
P(T_{3}=t_{3}|D=0)\times (1-\pi )\nonumber \\
& = \pi \left[\prod \limits_{i=1}^{2}
Se_{i}^{t_{i}}(1-Se_{i})^{(1-t_{i})} + (-1)^{t_{1}+t_{2}}
C_{+}\right]\\&\quad\times
[Se_{3}^{t_{3}}(1-Se_{3})^{(1-t_{3})}]\nonumber \\
&\quad +(1-\pi )\left[\prod \limits
_{i=1}^{2}Sp_{i}^{(1-t_{i})}(1-Sp_{i})^{t_{i}}+(-1)^{t_{1}+t_{2}}C_{-}\right]\nonumber
\\& \quad \times
[(1-Sp_{3})^{t_{3}}Sp_{3}^{(1-t_{3})} ].
\end{aligned}$$

Let $Y=(Y_{111},Y_{110},Y_{101},Y_{100},Y_{011},Y_{010},Y_{001},Y_{000})$, the observed data, and $\theta =(Se_{1},Sp_{1},Se_{2},Sp_{2},Se_{3},Sp_{3},\pi ,C_{+},C_{-})$, which represents the set of parameters in the model. According to (2), the likelihood function based on the observed data is:

3 $$\begin{aligned} L(\theta |Y)= & {} \prod \limits _{t_{1},t_{2},t_{3}}p_{t_{1},t_{2},t_{3}}^{Y_{t_{1},t_{2},t_{3}}}\nonumber \\= & {} \prod \limits _{t_{1},t_{2},t_{3}}\Big \{ \pi \Big [\prod \limits _{i=1}^{2}Se_{i}^{t_{i}}(1-Se_{i})^{(1-t_{i})}+(-1)^{t_{1}+t_{2}}C_{+}\Big ]\nonumber \\\times & \,{} \Big [Se_{3}^{t_{3}}(1-Se_{3})^{(1-t_{3})}\Big ]+(1-\pi )\Big [\prod \limits _{i=1}^{2}Sp_{i}^{(1-t_{i})}(1-Sp_{i})^{t_{i}}\nonumber \\+ &\, {} (-1)^{t_{1}+t_{2}}C_{-}\Big ] \times \Big [(1-Sp_{3})^{t_{3}}Sp_{3}^{(1-t_{3})}\Big ]\Big \}^{Y_{t_{1},t_{2},t_{3}}} \end{aligned}$$

To use the Bayesian method to estimate the vector of the parameters, $\theta$, we need to specify a prior distribution for $\theta$. Let $f(\theta )$ denote the prior distribution of $\theta$. The Bayesian method combines the prior information about $\theta$ with the data we have collected, and then uses the Bayes theorem to obtain an interpretable posterior distribution for $\theta$. We can use the median of the posterior distribution to estimate $\theta$. According to the Bayes theorem, the joint posterior distribution $f(\theta |Y)$ of the parameter $\theta$ given the observed data *Y* can be written as follows:

4 $$\begin{aligned} f(\theta |Y)=\frac{L(\theta |Y)f(\theta )}{\int L(\theta |Y)f(\theta )d\theta }=\frac{\mathcal {A}}{\mathcal {B}}, \end{aligned}$$

where

$$\begin{aligned} \mathcal {A}&= f(\theta )\prod \limits _{t_{1},t_{2},t_{3}}\Big \{ \pi \Big [\prod \limits _{i=1}^{2}Se_{i}^{t_{i}}(1-Se_{i})^{(1-t_{i})}+(-1)^{t_{1}+t_{2}}C_{+}\Big ]\\& \quad \times \Big [Se_{3}^{t_{3}}(1-Se_{3})^{(1-t_{3})}\Big ]+(1-\pi )\quad\times\Big [\prod \limits _{i=1}^{2}Sp_{i}^{(1-t_{i})}(1-Sp_{i})^{t_{i}}+(-1)^{t_{1}+t_{2}}C_{-}\Big ]\\& \quad \times  \Big [(1-Sp_{3})^{t_{3}}Sp_{3}^{(1-t_{3})}\Big ]\Big \}^{Y_{t_{1},t_{2},t_{3}}}, \end{aligned}$$

and

$$\begin{aligned} \mathcal {B} &= {} \underbrace{\int \int \cdots \int }_{9}f(\theta )\prod \limits _{t_{1},t_{2},t_{3}}\\&\quad\times\Big \{ \pi \Big [\prod \limits _{i=1}^{2}Se_{i}^{t_{i}}(1-Se_{i})^{(1-t_{i})}+(-1)^{t_{1}+t_{2}}C_{+}\Big ] \\ & \quad \times {} \Big [Se_{3}^{t_{3}}(1-Se_{3})^{(1-t_{3})}\Big ]+(1-\pi )\Big [\prod \limits _{i=1}^{2}Sp_{i}^{(1-t_{i})}(1-Sp_{i})^{t_{i}}+(-1)^{t_{1}+t_{2}}C_{-}\Big ]\\ \\ & \quad \times {} \Big [(1-Sp_{3})^{t_{3}}Sp_{3}^{(1-t_{3})}\Big ]\Big \}^{Y_{t_{1},t_{2},t_{3}}} \underbrace{dSe_{1}dSe_{2}\cdots dC_{+}dC_{-}}_{9} \end{aligned}$$

Consequently, the marginal posterior density function for any component in $\theta$, such as $Sp_{2}$, given the data, can be expressed as:

$$\begin{aligned} f(Sp_{2}|Y)&=\underbrace{\int \int \cdots \int \int }_{8}f(\theta |Y)dSe_{1} \\ & \quad \times dSe_{2}dSe_{3}dSp_{1}dSp_{3}d\pi dC_{+}dC_{-} \end{aligned}$$

By estimating the median of the margin distribution about $Sp_{2}$, denoted by $\widehat{{Sp}_{2}}$, we obtain a Bayesian estimate, $\widehat{{Sp}_{2}}$, for $Sp_{2}$.

## Procedure of the analysis

Here $T_{1}$,$T_{2}$, and $T_{3}$ denote the CM diagnostic scales (SSDS and ISDS) and ESD for detecting internal wind syndrome, respectively. The observed data can be represented by $Y=(69,19,7,12,32,9,5,51)$, as shown in Table 1. We denoted the proposed model as model (I). For comparison purposes, we also included the results obtained by the commonly used naive method, which assumed the ESD as the gold standard, and denoted this method as model (II). In the Bayesian analysis, a prior distribution for $\theta$, which was defined in the Bayesian model, had to be chosen.

### Selecting the prior distribution

A prior distribution for $\theta$ consisted of three sensitivities, three specificities, one prevalence rate, and two conditional covariances. Since the first six parameters have a range between 0 and 1, we chose a beta distribution $Beta (\alpha ,\beta )$ for each of them, where $\alpha$ and $\beta$ were hyper-parameters. We used the method proposed by Dendukuri [17] and Enøe et al. [27] to choose these hyper-parameter values by the priori moment information. According to the published literature describing the three diagnostic tests (SSDC, ISDS, and ESD) [2–5], the most probable value of the sensitivities of $T_{1}$ and $T_{2}$ for detecting internal wind syndrome was determined as 0.7, and we were 95 % sure that these sensitivities were less than 0.5. Thus, the prior distribution for the sensitivities $T_{1}$, $T_{2}$ was chosen to be the beta distribution, *Beta*(13.322, 6.281). For the specificities of the diagnostic scales $T_{1}$, $T_{2}$ for detecting internal wind syndrome, the most probable value was determined as 0.8, and we were 95 % sure that these specificities were less than 0.5. Therefore, the prior distribution for the specificities $T_{1}$ and $T_{2}$ was chosen to be the beta distribution, *Beta*(7.549, 2.637). The best guess value for the sensitivity of $T_{3}$ was 0.8, and the experts were 95 % sure that the sensitivity of $T_{3}$ was at least 0.7; hence, the prior distribution for the sensitivity of $T_{3}$ was chosen to be the beta distribution, *Beta*(48.283, 12.821). The best guess value for the specificity of $T_{3}$ was 0.85, and the experts were 95 % sure that the specificity of $T_{3}$ was at least 0.6; thus the prior distribution for the specificity of $T_{3}$ was chosen to be the beta distribution, *Beta*(10.657, 2.704). The uniform distribution on [0, 1] was used for the prior distribution of the internal wind prevalence rate. For the last two conditional covariances, $C_{+}$ and $C_{-}$, which measured the dependence of $T_{1}$ and $T_{2}$ among the diseased and non-diseased statuses, respectively, we have the following constraints: $(Se_{1}-1)(1-Se_{2})\le C_{+} \le min(Se_{1},Se_{2})-Se_{1}Se_{2}$ and $(Sp_{1}-1)(1-Sp_{2})\le C_{-} \le min(Sp_{1},Sp_{2})-Sp_{1}Sp_{2}$, respectively. Hence, we chose two uniform distributions for $C_{+}$ and $C_{-}$: $U((Se_{1}-1)(1-Se_{2}),(min(Se_{1},Se_{2})-Se_{1}Se_{2}))$ and $U((Sp_{1}-1)(1-Sp_{2}),(min(Sp_{1},Sp_{2})-Sp_{1}Sp_{2}))$.

### MCMC techniques for computing the posterior estimator

It was difficult to directly obtain the posterior estimator of each parameter through a numerical integration method in the Bayesian model. Since the joint posterior distribution $f(\theta \mid Y)$ was complicated and involved high-dimensional integral problems, which were often impossible to compute directly, we used the MCMC algorithm to draw a random sample from the joint posterior distribution. We then computed the sample median of the randomly drawn sample to estimate $\theta$ and its components of interest. In this study, the WinBUGS package was used to perform this MCMC process.

To use the MCMC technique in the Bayesian method, we specified the initial values of the model parameters, and the initial values were given as follows: $\pi =0.623,Se_{1}=0.748,Se_{2}=0.945,Se_{3}=0.850,Sp_{1}=0.844,Sp_{2}=0.883,Sp_{3}=0.935$, respectively. We also chose different initial values and obtained similar results. The numbers of iterations and burn-ins were determined by the convergence of the Markov chain in estimating the parameters by WinBUGS.

### Results and discussion

The sensitivity of the SSDC for internal wind syndrome (Table 2) was estimated as 0.687 by the Bayesian method in the absence of a gold standard, while the commonly used naive method, which uses the ESD as a gold standard, estimated the sensitivity of the SSDC as 0.673. The estimated sensitivity of the ISDS showed similar results. The Bayesian method estimated the specificity of the ISDS as 0.875, while the commonly used naive method estimated the specificity of the ISDS as 0.692. From these results, we can conclude that the commonly used naive method in CM for estimating the accuracy of diagnostic scales for this CM syndrome might be biased. Table 2 also shows the 95 % Bayesian confidence intervals for the sensitivity and specificity of the SSDC in detecting internal wind syndrome, which were (0.605,0.765) and (0.652,0.885), respectively. Similarly, the Bayesian confidence intervals for the sensitivity and specificity of the ISDS are also shown in Table 2.

**Table 2:** Accuracy of diagnostic scales (median) for internal wind syndrome factor in 204 ischemic stroke patients under different models

	Model I	Model II
$\hat{Se}_{1}$	0.687 (0.605,0.765)	0.673 (0.587,0.759)
$\hat{Se}_{2}$	0.884 (0.815,0.938)	0.894 (0.837,0.951)
$\hat{Se}_{3}$	0.813 (0.742,0.880)	N.A.
$\hat{Sp}_{1}$	0.776 (0.652,0.885)	0.659 (0.562,0.756)
$\hat{Sp}_{2}$	0.875 (0.739,0.968)	0.692 (0.597,0.787)
$\hat{Sp}_{3}$	0.922 (0.831,0.981)	N.A.
$\hat{\pi }$	0.648 (0.556,0.731)	0.554 (0.486,0.622)

As shown in Table 2, the respective Bayesian estimated sensitivities of the SSDC, ISDS, and ESD for diagnosing internal wind syndrome without a gold standard were as follows: $\hat{Se}_{1}=0.687$, $\hat{Se}_{2}=0.884$, and $\hat{Se}_{3}=0.813$. The respective estimated specificities of the SSDC, ISDS, and ESD for diagnosing internal wind syndrome in the absence of a gold standard were as follows: $\hat{Sp}_{1}=0.776$, $\hat{Sp}_{2}=0.875$, and $\hat{Sp}_{3}=0.922$. From these results, we concluded that the ISDS was more accurate than the SSDC in detecting internal wind syndrome. The Bayesian method also gave an estimate of $\hat{\pi }=0.648$ for the prevalence rate of internal wind syndrome. Hence, we concluded that the sensitivity and specificity of the ISDS were both higher than those of the SSDC when diagnosing internal wind syndrome in ischemic stroke patients. We also found that the sensitivity and specificity of the ESD for internal wind syndrome were also high, but not perfect.

To assess the sensitivity of our results to chosen prior distributions, we selected several different prior distributions for parameters in model (I). The posterior estimates under the chosen prior distributions for the parameters led to consistent results with the previous posterior estimates.

## Conclusion

After adjusting for imperfect gold standard bias, we found that both the sensitivity and specificity of the ISDS were higher than those of the SSDC for diagnosis of internal wind syndrome in ischemic stroke patients.

## Additional files

10.1186/s13020-016-0100-2 Approval document of ethics.
10.1186/s13020-016-0100-2 Ethics committee members.
10.1186/s13020-016-0100-2 Research Programme.
10.1186/s13020-016-0100-2 Informed consent.
10.1186/s13020-016-0100-2 WinBUGS code.

## Abbreviations

CM: Chinese medicine
SSDC: stroke syndrome differentiation diagnostic criterion
ISDS: ischemic stroke TCM syndrome diagnostic scale
ESD: expert syndrome differentiation
MCMC: Markov chain Monte Carlo
