# Supplementary figures and images for: Evaluation of the accuracy of diagnostic scales for a syndrome in Chinese medicine in the absence of a gold standard
Source: Chin Med. 2016 Jul 28;11:35. doi: 10.1186/s13020-016-0100-2 (PMC4964286; doi:10.1186/s13020-016-0100-2)

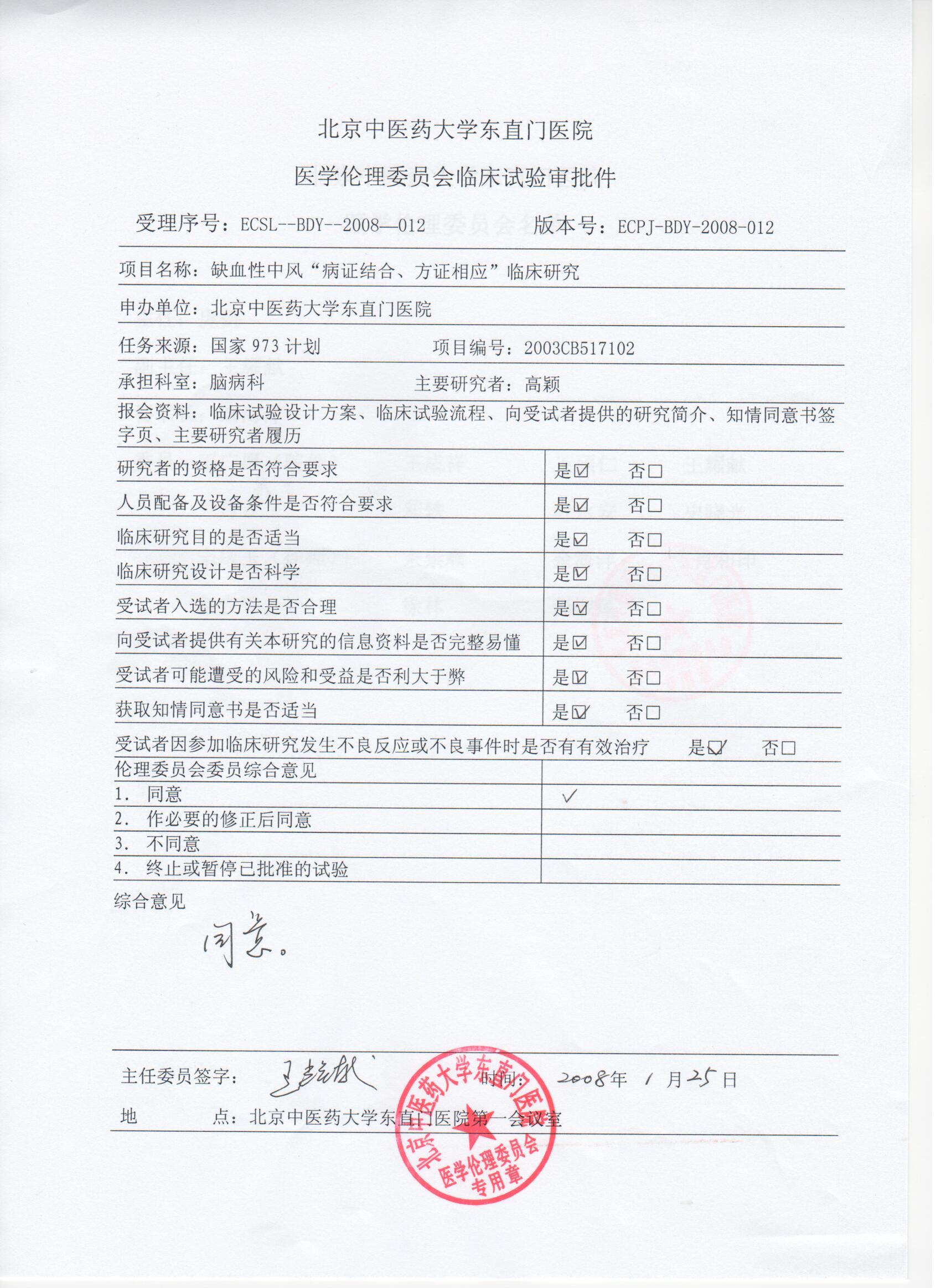

Supplement: Supplementary file 1 — 10.1186/s13020-016-0100-2 Approval document of ethics. [file 13020_2016_100_MOESM1_ESM.jpg]

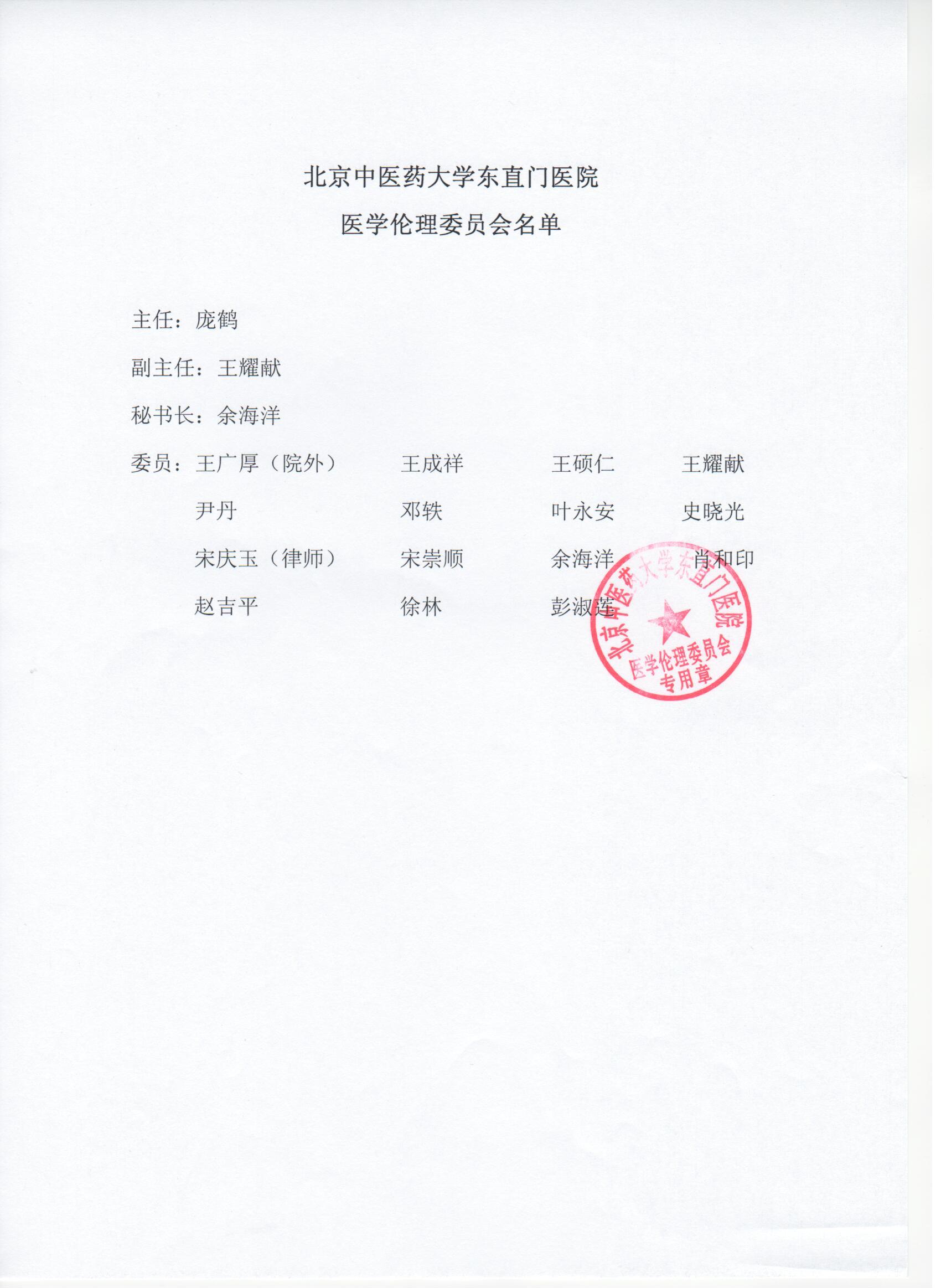

Supplement: Supplementary file 2 — 10.1186/s13020-016-0100-2 Ethics committee members. [file 13020_2016_100_MOESM2_ESM.jpg]
