# Supplementary material for: Evaluation of the accuracy of diagnostic scales for a syndrome in Chinese medicine in the absence of a gold standard
Source: Chin Med. 2016 Jul 28;11:35. doi: 10.1186/s13020-016-0100-2 (PMC4964286; doi:10.1186/s13020-016-0100-2)
Supplement: Supplementary file 3 — 10.1186/s13020-016-0100-2 Research Programme. [file 13020_2016_100_MOESM3_ESM.pdf]

国家重点基础研究发展计划（973 计划）

课题编号：2003CB517102

缺血性中风病证结合的诊断标准与疗效评价体系研究

— —缺血性中风“病证结合、方证相应”随机盲法、平行对照、多中心临床研究

方　案

课题承担单位：北京中医药大学东直门医院

2008.1

目 录

一、课题背景 1
二、研究单位 1
三、研究思路 1
四、研究内容 1
五、病例选择 2
六、研究方法 3
七、辅助检查 8
八、研究流程 8
九、不良事件与处理..................................9
十、受试者的依从性..................................10
十一、医学伦理要求..................................10
十二、质量控制与保证................................11
十三、数据管理......................................12
十四、统计分析......................................12

“缺血性中风病证结合的诊断标准与疗效评价体系研究”
——病证结合、方证相应研究方案

一、课题背景

“缺血性中风病证结合的诊断标准与疗效评价体系研究”是国家重点基础研究发展计划（973 计划）——“证候规范及其与疾病、方剂相关的基础研究”项目的课题之一，课题负责单位为北京中医药大学东直门医院，课题起止时间 2004.9——2009.8，课题分前两年和后三年两个阶段实施。第一阶段的研究工作已经通过了中期评估，第二阶段将进行缺血性中风“病证结合、方证相应”的多中心临床研究，以进一步诠释辨证方法新体系，验证缺血性中风证候诊断标准和疗效评价标准。

二、研究单位

课题负责单位：北京中医药大学东直门医院

课题负责人：　高颖

课题参加单位：天津中医药大学

广州中医药大学第二附属医院

河南中医学院第一附属医院

安徽中医学院第一附属医院

浙江中医药大学附属第一医院

湖南省中医药研究院（附属医院）

南京市中医院

辽宁省血栓病中西医结合医疗中心

佛山市中医院

三、研究思路

课题组在第一阶段研究工作的基础上，建立病证结合的缺血性中风疗效评价标准。在此基础上对形成的证候诊断标准进行前瞻性、多中心的临床验证；以缺血性中风急

性期患者为研究对象，在“证候要素、应证组合”思路指导下，针对证候要素的组合形式与特征，以“病证结合、方证相应”为指导原则，开展前瞻性、多中心、随机对照研究，以进一步完善辨证方法新体系。

四、研究内容

（一）标准验证:《中风病证候要素诊断量表》临床验证。

（二）临床研究: 缺血性中风“病证结合、方证相应”的临床研究。

缺血性中风急性期相关实验室指标的动态变化初步研究。

五、病例选择

（一）诊断标准

1. 疾病诊断

参照 2005 年卫生部疾病控制司、中华医学会神经病学分会颁布的《中国脑血管病防治指南（试行版)》。

2. 证候诊断

采用本课题组研究制定的《中风病证候要素诊断量表》

（二）纳入标准

1. 符合西医脑梗死诊断;
2. OCSP 分型中的前循环梗死;
3. 中医证候诊断符合“痰瘀证”、“痰热证”、“气虚血瘀证”、“阴虚证”四者之一;
4. NIHSS 分值≥5 分且≤22 分;
5. 发病在 72 小时以内;
6. 年龄≥35 岁且≤80 岁。

（三）排除标准

1. 短暂性脑缺血发作(TIA);
2. 脑出血或蛛网膜下腔出血;
3. OCSP 分型中的后循环梗死;
4. 超早期（3～6h 内）已接受溶栓治疗者;

5. 入院时存在意识障碍者;

6. 经检查证实由脑肿瘤、脑外伤、血液病等引起的卒中患者;

7. 因风湿性心脏病、冠心病及其他心脏病合并房颤，引起脑栓塞者;

8. 合并有肝、肾、造血系统、内分泌系统等严重疾病及骨关节病;

9. 精神障碍或严重痴呆;

10. 有卒中病史且遗留后遗症者;

11. 入院时伴重症感染者。

（四）受试者退出试验的条件及步骤

1. 研究者决定的退出

受试者退出试验是指已经入选的受试者在试验过程中出现了不宜继续进行试验的情况下，研究者决定该病例退出试验。

2. 受试者自行退出试验

根据知情同意书的规定，受试者有权中途退出试验，或受试者虽未明确提出退出试验，但不再接受用药及检测而失访，也属于“退出”(或称“脱落”)。应尽可能了解其退出的原因，并加以记录。

3. 无论何种原因，对退出试验的病例，应保留其病例报告表，并以其最后一次的检测结果结转为最终结果，对其疗效和不良反应进行全数据集分析。

（五）剔除病例标准

1. 严重违反纳入标准的病例。

2. 纳入后未曾用药或无任何可评价记录的病例。

六、研究方法

（一）整体设计

采用随机、盲法、平行对照、多中心临床试验设计方法。由北京中医药大学东直门医院临床研究中心负责随机化方案的设计。

（二）样本含量

根据国家“十五”科技攻关课题（2001BA701A12a）的研究成果，缺血性中风发病14天时综合治疗方案 NIHSS疗效比较中，综合治疗组（辨证论治组）有效率77.1%，西医组有效率60.1%，应用样本含量估算软件SASA1.0计算，取$\alpha=0.05$，$\beta=0.20$，

双侧差异性检验，每组至少需要观察87例患者，两组共174例。考虑20%的脱落率，确定样本含量为208例，治疗组和对照组各104例。

（三）对照设计

试验组与对照组采用1：1对照设计。

（四）随机设计

采用分层区组随机化方法。按中心进行分层，选取合适段长。借助SAS统计软件PROC PLAN过程语句，给定种子数，产生208例受试者所接受处理的随机安排，即列出流水号为001－208所对应的治疗分配（即随机编码表）。整个过程由10所医院协同完成。

（五）盲法设计

1、设盲方法

依据随机编码表，双盲试验的设盲工作由临床研究负责单位和统计人员共同完成，采用两级设盲法，第一级为编码对应的三处理组代号，第二级为代号对应的处理组。

2、应急信件的准备

（1）应急信件的准备由专门的软件系统制作，信封上印有***临床试验的应急信件、药品编号等。如果拆阅，需注明拆阅者，拆阅日期，原因等，并在病例报告表中记录。

（2）信封内印有该受试者的服药信息、处理方法及应立即汇报的单位和地址。应急信件准备完后，随药物发往各个中心，在试验结束后统一收回。

3、按处理编码对药物进行包装和编号

根据已形成的处理编码将相应的药物分成2组，即A、B组。将同样包装的中风1号方、2号方、3号方和4号方按组别分别放置。

4、编盲记录

全部药品编码过程由编盲者书写成文件形式，即编盲记录，作为该临床试验的文件之一保存。其内容包括：研究药品的准备，药品的包装，用法，储存要求，药品发放办法，随机处理编码的产生，应急信件，盲底的保存，揭盲的保存，揭盲的规定和各个中心受试患者的药物编号等。

5、盲底保存规定

全部处理编码所形成的盲底连同产生随机数的初值、区组的长度等参数，密封后一式两份分别交主要研究者和统计学家两处妥善保存，试验期间盲底不得拆阅。如果

发生了任何非规定情况所致的盲底泄漏，并影响了该试验结果的客观性，则该试验将被视作无效。

6、紧急情况下个别案例揭盲规定

出现严重不良事件、或死亡、或需紧急抢救时，由分中心的负责研究者报告主要研究者，决定是否需拆开应急信件。应急信件一旦被拆阅，该编号受试者将退出试验，研究者应将原因记录在病例报告表中。所有应急信件在试验结束后随病例报告表一起收回，以便试验结束后盲态审核。

7、揭盲规定

病例收集结束，建立数据库并锁定数据后进行两级揭盲，先明确各编号对应组代号进行统计分析，统计分析完成后再明确各代号对应的处理。

（六）病例分配

采用多中心临床研究方法，研究中心共10家，所观察病例数均按照试验组与对照组1：1计算，10家研究中心共观察208例符合纳入标准的病例，试验组与对照组病例数均为104例。各临床研究中心应严格按本方案规定的各项标准选择病例。

（七）药品来源

试验组与对照组均提供中风1号方、中风2号方、中风3号方、中风4号方共计4种中药免煎颗粒剂。采用一级设盲，双模拟。

试验组：中风1号方（化痰通络汤），中风2号方（星蒌承气汤+化痰通络汤模拟方），中风3号方（益气化瘀汤+化痰通络汤模拟方），中风4号方（育阴熄风汤+化痰通络汤模拟方）

对照组：中风1号方（化痰通络汤），中风2号方（化痰通络汤+星蒌承气汤模拟方），中风3号方（化痰通络汤+益气化瘀汤模拟方），中风4号方（化痰通络汤+育阴熄风汤模拟方）

剂型采用免煎颗粒，由三九集团生产。

1. 化痰通络汤

法半夏6g 生白术12g 天麻12g 紫丹参30g 三七3g 香附10g 酒大黄3g

2. 星蒌承气汤

全瓜蒌30g 胆南星6g 生大黄6g（后下） 芒硝10g（冲服） 丹参30g

3. 益气化瘀汤

生黄芪 30g　全当归 10g　　桃仁 10g　 草红花 6g　赤芍 10g　柴胡 6g　　川芎 6g

4. 育阴熄风汤

生地黄 20g　山萸肉 12g　　钩藤 30g　　天麻 12g　　丹参 30g　 白芍 10g

化痰通络汤、星蒌承气汤、益气化瘀汤、育阴熄风汤方剂组成来源于全国中医急症中风病科研协作组和全国中医内科学会中风病学组制订的《中风病中医诊疗规范》（1988 年）及国家“七五”科技攻关课题关于“中风病系列方药”的研究成果。经专家论证调整后在国家“十五”攻关课题“中风病急性期综合治疗方案研究”中作为治疗方案的核心内容经多中心的随机对照临床研究对其安全性和有效性进行了评价。

（八）治疗方案

1. 内科基础治疗

主要参考 2005 年卫生部疾病控制司、中华医学会神经病学分会《中国脑血管病防治指南（试行版）》制定的脑梗死治疗方案。

（1）抗血小板药物：肠溶阿司匹林(拜阿司匹灵)100mg／次，口服，每日一次。如出现不良反应，用其他口服抗血小板制剂替代。

（2）注射剂：使用哈尔滨三联药业有限公司生产的注射用盐酸川芎嗪（粉针剂），入组后第 1-14 天使用，120 毫克加入 5%葡萄糖注射液或 0.9%氯化钠注射液 250～500 毫升中，静脉滴注，每日 1 次，连续使用 14 天。

（3）根据病情需要可按照《中国脑血管病防治指南（试行版）》的规定，酌情使用降纤、抗凝治疗和神经保护剂。

（4）入组至发病第 21 天禁止使用研究方案以外的药物，如中药汤剂、中药注射剂、治疗中风病的中成药等。入组至发病第 14 天禁止使用针灸治疗。

（5）其它治疗主要包括：血压的调控、血糖的调整、感染的治疗等。

（6）康复和护理：对患者进行规范的康复训练和护理。

2. 辨证论治

无论试验组或对照组，判断证候后，均根据证候要素组合的形式与特征，以“病证结合、方证相应”为指导原则，调整处方。“痰瘀证”选用中风 1 号方，“痰热证”选用中风 2 号方，“气虚血瘀证”选用中风 3 号方，“阴虚证”选用中风 4 号方。按照证候所对应方剂，给予 4 种免煎颗粒剂中的一种。每日 1 剂，分 2 次冲服。

所用免煎颗粒剂统一由课题负责单位免费提供。

（九）证候诊断原则：

采用本课题组研究制定的《中风病证候要素诊断量表》，达到证候要素诊断分值（≥10 分），即为该证候要素诊断成立。本量表为诊断量表，各证候要素的总分不同，故各证候要素得分之间的差异不能做为证候要素轻重程度的判断依据。如多个证候要素诊断同时成立，请结合观察医师临床经验（不是按各证候要素得分高低），判断其主次并排序，从而确定证候诊断符合“痰瘀证”、“痰热证”、“气虚血瘀证”、“阴虚证”四者之中哪个证候，纳入病例并据证选方换方。

注意事项：1.若治疗过程中出现不在上述考虑范围之内的证候，观察医师应对该证候做详细描述，并维持原方继服。2.若“痰热证”患者连续应用中风 2 号方免煎颗粒剂 3 天后，仍大便不通者，可以按西医治疗常规应用泻药或清洁灌肠，如：果导片（酚酞）、开塞露、甘油栓剂等。

（十）疗程：入院当天至发病第 21 天。（如遇特殊情况提前出院的，按照原方案门诊治疗观察至发病第 21 天）

（十一）随访：发病第 28 天、60 天、90 天进行随访。

（十二）研究用药管理制度

研究前管理：研究前由课题负责单位准备免煎颗粒剂，并按盲法设计要求包装药品，分发给各临床研究中心 A 组、B 组共 2 箱药，每箱药均提供中风 1 号方、中风 2 号方、中风 3 号方、中风 4 号方共计 4 种免煎颗粒剂。

研究期管理：建立研究药品管理制度。观察医师在患者签署知情同意书后，按入组顺序确定随机号，拆开随机信封，确定组别，并根据中医辨证确定患者所服用的方剂。药品专柜上锁，室温保存。每次发药时药品管理员于药品发放登记本上记录发药日期、患者姓名、随机号、组别、颗粒剂名称、给药数量，并签名。

供药方式：初次供药将根据以往研究工作估算用量，邮寄给各临床研究中心，如课题进行中各临床研究中心 2 个药箱 4 种免煎颗粒剂中任何 1 种剩余 1/3 药量时，请根据研究进度，及时联系课题负责单位，以决定是否需要补发药物。

研究后管理：每个临床研究中心将剩余药品集中返还课题负责单位。

（十三）临床评价内容

1. 观察时点

入院当天、发病第 3 天、第 7 天、第 14 天、第 21 天。

发病第 28 天、第 60 天、第 90 天进行随访。

2. 观察与评价指标

意识评分（Glasgow 昏迷评分量表）

神经功能缺损改善情况（NIHSS）

日常生活活动能力改善情况（Barthel 指数）

病残程度（改良 Rankin 量表）

智能评分（MMSE）

中风病患者症状学问卷

中风病患者临床疗效自评估

中医证候的判断（《中风病证候要素诊断量表》、《中风病辨证诊断标准》、三位专家判断）

安全性指标

七、辅助检查

（一）动态检测指标

1. 检测时间：发病 3 天内、第 7 天、第 14 天各检测一次。

2. 检查单位：统一由课题负责单位检测。

（二）血凝、D-二聚体

1. 检查时间：发病 3 天内检测一次。

2. 检查单位：由各研究单位自行检测。

（三）头颅 CT/MRI

1. 检查时间：发病 3 天以内检查，如遇特殊情况可延迟。

2. 检查单位：由各研究单位自行检测。

八、研究流程

（一）入组前的工作

1. 初步筛选：

（1）确定是否为脑梗死;

（2）询问患者年龄;

（3）确定发生脑梗死的时间;

（4）判断脑梗死 OCSP 分型;

（5）评估脑梗死严重程度（NIHSS 分值);

（6）确定是否为中风病的“痰瘀证”、“痰热证”、“气虚血瘀证”、“阴虚证”四者之一;

2. 签署知情同意书。

（二）入组当天的工作

1. 确定随机号及组别;

2. 填写病历记录;

3. 采集一般资料;

4. 判断证候、分发药物;

5. 进行辅助检查。

（三）入组后的工作

1. 判断证候、分发药物;

2. 填写病例报告表相关内容;

3. 按时点进行辅助检查;

4. 入组 3 天内将一般资料传真给课题负责单位;

5. 将保存待测的纳入患者血清标本按要求大约每 3 个月包装快递 1 次;

6. 发病第 28 天、第 60 天、第 90 天随访。

九、不良事件与处理

不良事件是病人或临床试验的受试者接受一种药品后出现的不良医学事件，但不一定与治疗有因果关系。也包括研究开始前存在的疾病发病次数和严重程度的增加。严重不良事件是指临床试验过程中发生需住院治疗、延长住院时间、伤残、影响工作能力、危及生命或死亡、导致先天畸形等事件。

（一）记录与报告

研究者应向患者说明，要求患者如实反映用药后的病情变化。医生避免诱导性提

问。在观察疗效同时，注意观察不良反应。无论不良反应或不良事件是否与本研究用药相关，均应详细记录，包括不良反应出现时间、症状、体征、程度及发作频度、持续终止时间、实验室检查指标、处理方法与结果、经过、随访时间等。并应详细记录合并用药的情况，以便分析不良反应与试验药物的相关性。是否需要治疗，如需要，请记录所给予的治疗。研究者判断不良事件是否与应用试验药品有关。随访选择住院、门诊、家访、电话等形式。

如果发生严重不良事件，研究者必须在 24 小时之内或不迟于第二个工作日向临床研究负责单位报告。研究者要在报告上签名并注明日期，在原始资料中记录何时、以何种方式、向谁报告了严重不良事件。

（二）紧急破盲信封的拆阅与处理

随药品下发的紧急破盲信封只有在该名受试者发生严重不良事件，需立即查明所服药品的种类，才由研究单位的主要研究者拆阅，即紧急揭盲。一旦揭盲，该受试者将被中止试验，并作脱落病例处理，同时将处理结果通知监查员，并在病例报告表中详述理由、日期并签字。

（三）病人的处理

发现不良反应时，研究者可根据病情采取必要的处理措施，如：调整试验用药剂量、暂时中断用药、永久性停药等。出现严重不良事件，承担临床研究的单位须立即采取必要处理措施，保护受试者安全。所有不良事件都应当追踪调查，详细记录处理经过及结果，直到得到妥善解决或病情稳定，若化验异常者应追踪至恢复正常。追踪随访方式可以根据不良反应的轻重选择住院、门诊、家访、电话、通讯等多种形式。

十、受试者的依从性

用药依从性（%）=（处方量-漏服量）/处方量×100%

十一、医学伦理要求

本临床试验将参照赫尔辛基宣言（1996 年版）和我国有关新药临床试验研究规范、法规进行。

临床研究开始前制定研究方案，本方案经专家论证后由课题组报临床研究负责单

位伦理委员会批准后方可开始本项研究。

十二、质量控制与保证

（一）本课题组成立专家指导小组，进一步完善研究方案各环节实施细则，提高临床可操作性，对研究中遇到的问题随时进行指导;

（二）成立课题领导小组，由课题负责单位负责人牵头，各参加单位负责人组成，主要负责课题组织、实施、管理，各临床研究中心设本单位联系人 1 名;

（三）编制研究者工作手册，制定“病证结合、方证相应”研究临床信息采集的标准规范，以保证临床信息采集的客观性，并确保本课题在严格的盲态下进行;

（四）各临床研究中心应相对固定研究人员，尽量避免在课题执行中再更换，对参加临床研究人员进行严格统一培训，要求熟练掌握中风病的临床诊治，指标的观察，标本的留取，及评价量表的使用方法，进行考核与一致性检验。即先后由两位研究者单独对同一患者进行调查，计算两人临床意见一致符合率，即 Kappa 值，以保证临床操作的一致性;

（五）严格管理观察表，研究者应按要求如实、详细、认真记录各项观察内容，以确保病例报告表内容真实、可靠和完整。原始数据不得涂改，如确有笔误，修改时需保持原有记录清晰可见，改正处需研究者签名并注明日期;

（六）定期随访，控制病例脱落，在方案实施中保证医师和患者的依从性，针对可能发生的脱落，积极采取措施，控制病例脱落率在 20%以内。患者出院时，由主管医师进行健康宣教，预约复诊时间，定期进行随访;

（七）设立质量控制小组，由课题负责单位派遣临床监查员定期对各临床研究中心现场监查访问，抽样核对原始资料，并就工作中的具体问题定期召开会议，及时以监查报告和课题简报的方式反馈信息，提高监查质量;

（八）设立数据管理中心，加强数据管理，制定数据录入与核查的操作规则，研究过程中及时将各临床研究中心采录的试验数据加以整理、检查、归档，发现问题及时通报、解决，以保证数据的准确性和真实性。

十三、数据管理

（一）数据传真

每位患者纳入后 3 天内，观察医师将患者《病例报告表 I》第 2 页（即入院当天的一般资料、病史、诊断、治疗史）复印后传真至课题负责单位北京中医药大学东直门医院，以保证信息及时汇总分析。传真号 010-84013351。

（二）病例报告表的填写与移交

研究者根据受试者的原始观察记录，将数据及时、完整、正确、清晰地载入病例报告表。每个入选病例必须完成病例报告表。完成的病例报告表由临床监查员审查后，移交数据管理员，进行数据录入与管理工作。

（三）数据的录入与修改

数据录入与管理由数据管理员负责。数据管理员应与主要研究者一起，按病例报告表中各指标数值的范围和相互关系拟定数据范围检查和逻辑检查内容，并编写相应的计算机程序，在输入前控制错误数据输入，所有错误内容及修改结果应有记录并妥善保存。为保证数据的准确性，应由两个数据管理员独立进行双份录入并校对。对病例报告表中存在的疑问，数据管理员将产生疑问解答表(DRQ)，并通过临床监查员向研究者发出询问，研究者应尽快解答并返回，数据管理员根据研究者的回答进行数据修改、确认与录入，必要时可以再次发出疑问解答表(DRQ)。

（四）数据锁定

在盲态审核并确认建立的数据库正确后，由课题负责单位人员、统计分析人员对数据进行锁定。锁定后的数据文件不再做改动。数据锁定之后发观的问题，经确认后在统计分析程序中进行修正。

（五）揭盲和数据处理

在研究数据全部录入并锁定后，由保存盲底的工作人员做第一次揭盲，并将数据库交统计分析人员按统计计划书要求进行统计分析。完成统计分析后，再由课题负责单位工作人员进行第二次揭盲，最后由统计分析人员写出统计分析报告，交课题负责单位研究人员写出研究报告。

十四、统计分析

（一）统计分析数据集

全分析集 (Full Analysis Set, FAS)：根据 ITT 原则，所有经随机化分组，并至少使用一次药物的全部病例的数据构成本研究的全分析集（FAS），FAS 是本次研究的主要分析数据集。FAS 中疗效相关部分的缺失数据将采用之前最后一次观测数据结转的方法进行补充。

符合方案集 (Per-Protocol Set, PPS)：所有符合试验方案、依从性好、试验期间未用禁止药物、完成主要有效性指标评价，使用试验用药数量在 80%—120%者的数据，构成符合方案集（PPS），PPS 是本次疗效评价的次要数据集。

安全性数据集（Safety Set,SS）：指使用过试验药物并至少有一次安全性评价记录的病例数据。

（二）统计分析计划

描述性统计分析，定性指标以频数表、百分率或构成比描述；定量指标符合正态分布的以均数、标准差来描述，符合偏态分布的用中位数(P50)，第 25 百分位数(P25)，第 75 百分位数（P75）来描述。

两组对比分析，定性资料采用 X^2 检验（Chi-Square test），Fisher 精确概率法，Wilcoxon 秩和检验。三组之间的两两比较采用卡方分割法(Partitions of X^2 method)。

定量资料符合正态分布用 T 检验(组间进行方差齐性检验，以 0.05 作为检验水准，方差不齐时选用 Satterthwaite 方法进行校正的 t 检验)，不符合正态分布用 Wilcoxon 秩和检验。

假设检验统一使用双侧检验，给出检验统计量及其对应的 P 值，以 P≤0.05 作为有显著性统计学意义，以 P≤0.01 作为有高度显著性统计学意义。

证候量表验证使用基线证候数据，取 3 个专家当中 2 名或 2 名以上的一致意见作为证候要素存在与否的临床判断，并以此为近似“金标准”。使用 SPSS13.0 软件进行统计分析，通过四格表的方式记录《中风病辨证诊断标准》和《缺血性中风证候要素诊断量表》的诊断数据，分别计算两个量表的灵敏度、特异度、Youden 指数和正确率。

同时绘制量表诊断 ROC（Receiver Operating Characteristic）曲线，计算曲线下面积。ROC 曲线即受试者工作特性曲线，是用真阳性率（灵敏度）和假阳性率（1-特异度）作图所得曲线；常用于表示灵敏度和特异度之间的关系。
