# Supplementary material for: Evaluation of the accuracy of diagnostic scales for a syndrome in Chinese medicine in the absence of a gold standard
Source: Chin Med. 2016 Jul 28;11:35. doi: 10.1186/s13020-016-0100-2 (PMC4964286; doi:10.1186/s13020-016-0100-2)
Supplement: Supplementary file 4 — 10.1186/s13020-016-0100-2 Informed consent. [file 13020_2016_100_MOESM4_ESM.pdf]

课题编号 2003CB517102　　中心号□□　　随机号□□□

缺血性中风“病证结合、方证相应”临床研究
患者知情同意书

尊敬的患者：

我们请您参加一项临床研究，将动态观察缺血性中风急性期患者服用中药后中医证候的演变过程和规律。此项临床研究是“缺血性中风病证结合的诊断标准与疗效评价体系研究”的一部分，得到国家重点基础研究发展计划（973 计划）的资助，课题负责单位为北京中医药大学东直门医院，经研究负责单位伦理委员会批准后进行。

如果您决定参加本研究，医生将检查您是否符合参加本研究的条件，询问您的健康情况和有关疾病的情况，医生还将对您进行体格检查。如果您符合本研究的入选条件，您将被随机分配到两个研究组中，两组均进行内科基础治疗，以保证您得到目前对脑血管病有效的西医治疗。在此基础上，给予不同的中药治疗，采用一级设盲双模拟方法，由观察医师判断您的中医证候，根据病情变化随时调整用方，选用适合您证候的中药免煎颗粒剂，每日 1 剂，分两次冲服，疗程 21 天。此方案是经多年研究而优化的治疗方案，所用方剂经多年临床验证安全有效。

您参加本研究完全是自愿的。我们希望您能坚持完成本研究。如果您在任何时候因为任何原因退出本研究，请与您的医生联系，并完成最后一次就诊时所要求回答的问题，以便医生能完成最后一次就诊要求的所有检查项目。如果您决定退出本研究，将不会影响您与医生的关系，医生将根据您的病情和患者至上的原则对您进行合理的治疗。

参加本研究您将得到免费的中药免煎颗粒剂治疗及相关实验室检查。与本研究有关的机体损伤目前尚未发现，如果发生上述情况，您将得到及时的相应治疗措施，但这种损伤必须是与本临床研究有关的。

签署了这份知情同意书，即表示您同意参加本研究。

有关您身份的记录都是保密的，只有您的医生清楚。您的临床研究资料将记录在病例报告表上。您的姓名不会出现在任何研究报告和公开出版物中。

我已仔细阅读了以上内容，并且我的医生给我作了完整的解释，对于本研究的意义、目的和具体方法我已了解。我自愿参加本研究，并愿意按研究方案要求与医生合作，完成本次研究，我也同意有关方面对照我的原始记录检查核对收集的资料。

患者签名：　　　　　　　　　　　　年　月　日

我已详细向患者介绍了整个研究过程，并告知患者在研究过程中的风险和获益情况。

医生签名：　　　　　　　　　　　　年　月　日

联系电话：
